# 3D HRCT Analysis of Supratubal Recess Volume in Chronic Otitis Media: A Case-Control Study

**DOI:** 10.3390/medicina62071324

**Published:** 2026-07-09

**Authors:** Mehmet Murat Günay, Meltem Yıldırım Erol, Nazlı Nur Besler, Sibel Alicura Tokgöz, Halime Çomruk, İlker Akyıldız, Murad Mutlu, Samet Özlügedik

**Affiliations:** 1 Department of Otolaryngology-Head and Neck Surgery, Etlik City Hospital, 06170 Ankara, Turkey; n.aydinc24@gmail.com (N.N.B.); salicura@yahoo.com (S.A.T.); ilkerakyildiz@yahoo.com (İ.A.); muradmutlu78@yahoo.com (M.M.); sametozlugedik@yahoo.com (S.Ö.); 2 Department of Radiology, Etlik City Hospital, 06170 Ankara, Turkey; meltemyildirim51@gmail.com (M.Y.E.); dal_halime@hotmail.com (H.Ç.)

**Keywords:** supratubal recess, high-resolution CT, pneumatization, chronic otitis media, age effects

## Abstract

*Background and Objectives:* This study investigated the association of supratubal recess (STR) volume with chronic otitis media (COM), mastoid pneumatization, and age using three-dimensional (3D) high-resolution computed tomography (HRCT). *Materials and Methods:* A retrospective analysis was performed on 152 adult patients who underwent surgery for COM and 35 control subjects (70 ears) who underwent cochlear implantation. STR dimensions (width, length, and height) were measured on preoperative HRCT, and STR volume was calculated using Horos software (v4.0.0, macOS) through manual segmentation validated by two blinded radiologists. STR volume was analyzed in relation to mastoid pneumatization type (pneumatized, diploic, and sclerotic), age, and disease subgroup (chronic suppurative otitis media [CSOM] vs. chronic non-suppurative otitis media [CNSOM]). *Results:* STR volume was significantly higher in pneumatized mastoids compared with diploic and sclerotic types (*p* < 0.001), with sclerotic mastoids showing the greatest reduction. Univariate analysis showed no significant baseline STR volume differences among CSOM, CNSOM, and controls (mean volumes ± SD: 12.46 ± 6.39, 12.51 ± 7.19, and 13.18 ± 4.90 mm^3^; *p* = 0.751). However, multivariable regression indicated that both CSOM (β = 0.220, *p* = 0.002) and CNSOM (β = 0.416, *p* < 0.001) were positively associated with STR volume compared to controls, while diploic (β = −0.519, *p* < 0.001) and sclerotic (β = −0.665, *p* < 0.001) mastoids were associated with significantly lower STR volumes compared to pneumatized mastoids. Age showed a positive trend without reaching statistical significance (*p* = 0.053), and sex was not a significant predictor (*p* = 0.385). Reliability analyses demonstrated good intraobserver agreement (ICC = 0.88) and moderate-to-good interobserver consistency. *Conclusions:* STR volume is strongly influenced by mastoid pneumatization and independently associated with disease type, whereas age-related changes in adulthood and sex are negligible. 3D HRCT volumetry offers a deeper anatomical understanding of the supratubal recess and may facilitate more comprehensive anatomical and radiological evaluations.

## 1. Introduction

The supratubal recess (STR), also known as the anterior epitympanic recess, is a dome-shaped anatomical structure located anterior to the malleus. The STR is bounded by critical anatomical structures, including the tegmen tympani superiorly, the cochleariform process and tensor tympani fold inferiorly, the cog posteriorly, and the zygomatic root anteriorly. Medially, it is adjacent to the tympanic segment of the facial nerve and the geniculate ganglion, while laterally it is bordered by the scutum [1,2]. It develops in utero, is present at birth, and continues to grow throughout childhood until the age of 18 [3,4,5].

The supratubal recess (STR) represents one of the most challenging regions in otologic surgery, particularly for cholesteatoma removal, as it frequently harbors residual disease and contributes to high recurrence rates [6,7,8]. Owing to its proximity to critical structures, preoperative evaluation of STR anatomy using high-resolution computed tomography (HRCT) is recognized as essential for safe and precise surgical intervention [9]. Comprehensive knowledge of STR morphology and the factors influencing its variation is crucial, not only for ensuring safe and effective surgical management but also for understanding the anatomical basis of middle ear disease. Previous studies have demonstrated a positive correlation between mastoid pneumatization and STR dimensions, particularly length and height [5]. The literature on age-related changes in adulthood is limited; however, Hong et al. reported a decrease in STR width in individuals over 18 years of age, whereas STR length and height remained stable [4]. Despite these findings, comprehensive volumetric assessments of the STR in relation to chronic otitis media, mastoid pneumatization, and age in adulthood remain scarce.

This study aimed to evaluate the influence of mastoid pneumatization, age, and otologic disease type (chronic suppurative otitis media [CSOM] vs. chronic non-suppurative otitis media [CNSOM]) on STR volume using three-dimensional (3D) HRCT volumetric analysis.

## 2. Material and Methods

### 2.1. Study Design and Patient Selection

This study was approved by the Ethics Committee of Etlik City Hospital (approval number: AEŞH-BADEK-2024-562; date: 6 June 2024). All study procedures were conducted ethically and in accordance with the requirements of the Helsinki Declaration of 1975, as revised in 2008. Patient consent was waived in this study due to its retrospective -observational design, non-invasive nature involving only chart review. Re-contacting participants is not feasible and all data were analyzed and reported in anonymized/coded form, posing no additional risk to participants.

A retrospective analysis was conducted on 152 adult patients who underwent surgery for chronic otitis media (COM) and 35 adult patients who underwent cochlear implantation between November 2022 and May 2024 at a tertiary referral center. Patients were categorized into three groups:

- Group 1 (CSOM): Patients with tympanic membrane perforation and persistent otorrhea for 3 months or longer.
- Group 2 (CNSOM): Patients with tympanic membrane perforation but no otorrhea for at least 3 months.
- Group 3 (Control): Cochlear implant recipients with no history of chronic otitis media or otologic surgery, serving as the control group.

Cochlear implant recipients were selected as the control group because they routinely undergo preoperative HRCT. Control group eligibility required: (1) a normal tympanic membrane on otoscopic examination; (2) no history of chronic otitis media, otologic surgery, or temporal bone fracture; (3) normal middle ear physiology confirmed by tympanometry; and (4) normal middle ear anatomy (without congenital anomalies) on temporal bone HRCT. Between November 2022 and May 2024, 47 adult cochlear implantations were performed in our clinic, and 35 patients who met the control group eligibility criteria were included in the study. As both ears of these patients met these requirements, data from both ears were included in the analysis, resulting in a total of 70 ears from 35 patients.

The contralateral ear in the CSOM and CNSOM groups was not chosen as a control for two main reasons:

(a) a considerable proportion of contralateral ears had a history of surgery (27% in the CSOM group and 11% in the CNSOM group); and

(b) even among patients without a surgical history, many had concomitant pathologies in the contralateral ear (32% in the CSOM group and 19% in the CNSOM group).

This study specifically examines non-cholesteatomatous COM. While STR involvement in cholesteatoma has been well-documented in the literature, we excluded cholesteatomatous cases due to their distinct pathophysiology and to prevent measurement confounding. Attic cholesteatomas frequently erode or remodel STR anatomy, which would preclude accurate volumetric assessment using our methodology. This deliberate exclusion ensures internal validity for non-cholesteatomatous COM evaluation.

**Figure 1:**
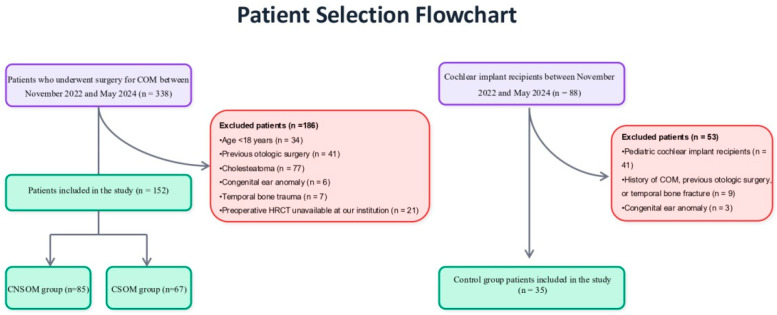
Patient selection flow diagram illustrating the inclusion and exclusion criteria for the study and control groups.

The dataset was complete, and no missing values were present due to our strict inclusion and exclusion criteria. The patient selection process, including the inclusion and exclusion criteria, is illustrated in Figure 1.

### 2.2. Imaging and Measurements

All patients underwent preoperative HRCT using a 64-detector row scanner (GE Revolution Evo, 128-slice CT, General Electric Medical Systems, Milwaukee, WI, USA). Imaging parameters included a slice thickness of 0.625 mm, pitch of 0.531 mm, a rotation time of 1 s, and 100 kVp. Images were displayed using a window center of 700 HU and a window width of 4000 HU. STR volume was measured using Horos software (v4.0.0, macOS) through a manual segmentation technique, performed independently by two experienced radiologists blinded to the study groups. To ensure measurement reliability, the original radiologist repeated all measurements three months after the initial assessment to evaluate intraobserver variability. Measurements obtained by the second radiologist were used to assess interobserver variability, and intraclass correlation coefficients (ICCs) were subsequently calculated.

**Figure 2:**
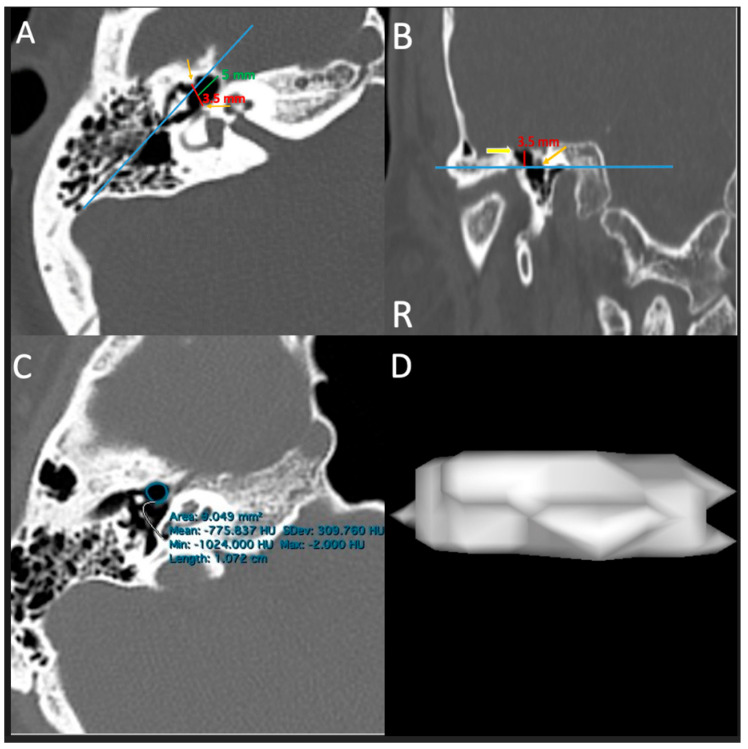
Axial HRCT images demonstrating supratubal recess (STR) measurements and 3D volumetric reconstruction. Axial HRCT scan through the right STR, showing measurements of length (green line) and width (red line) of STR. STR length: the deepest anterior-to-posterior diameter, parallel to incudomalleolar axis (blue line), STR width: distance between medial and lateral borders of cog (thin orange arrows) (**A**). Measurement of STR height on a coronal HRCT scan, from tegmen tympani (thick yellow arrow) to the cochleariform process, adjacent to the tensor tympani muscle (thin orange arrow), R: Right (**B**). The figure illustrates the measurement of supratubal recess volume using a manual segmentation protocol. In this method, markings are made on serial CT images to determine the volume, followed by 3D reconstruction to calculate the volume in mm^3^. Diagram of the morphometric schematic representation of STR volume in a pneumatized mastoid (**C**,**D**).

Supratubal recess dimensions were defined as follows:

- STR Width (STR-W): Defined as the distance between the most lateral and inferior point to the most medial and inferior point of the cog in the axial plane.
- STR Length (STR-L): Represented by a line parallel to the axis of the incudomalleolar articulation, extending from the most anterior point of the STR to the line passing through the cog in the axial plane.
- STR Height (STR-H): Defined as the superior-to-inferior (SI) distance from the tegmen tympani to the cochleariform process and the most superior point of the tympanic orifice of the Eustachian tube in the coronal plane (Figure 2) [4,9,10].

Supratubal recess volume measurement:

- The volume of the supratubal recess was determined using a manual segmentation protocol. In this method, the STR was identified on axial images and confirmed via multiplanar reconstructions. The anatomical boundaries were defined as follows: the tegmen tympani superiorly; the cochleariform process and superior margin of the Eustachian tube orifice inferiorly; the cog posteriorly; the furthest visible extension anteriorly; and the surrounding bony margins medially and laterally. Manual segmentation was performed by tracing the visible STR margins on serial axial CT slices. The segmented areas were multiplied by the slice thickness and summed to calculate the total volume. A three-dimensional model was generated using Horos software, and volumes were recorded in mm^3^ (Figure 2) [11]. In poorly pneumatized or partially sclerotic cases, all planes were evaluated simultaneously. Only the clearly identifiable pneumatized portion within these predefined borders was segmented, without incorporating any extrapolated or estimated volumes.

**Figure 3:**
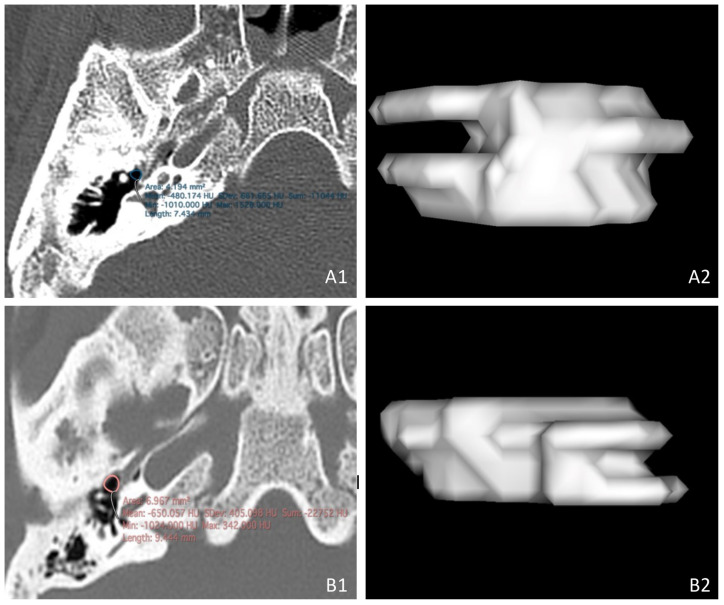
Axial HRCT image showing the measurement of the right STR in a sclerotic mastoid (**A1**); corresponding 3D volumetric reconstruction of the STR, with a measured volume of 3.9 mm^3^ (**A2**). Axial HRCT image showing the measurement of the right STR in a diploic mastoid (**B1**); corresponding 3D volumetric reconstruction of the STR, with a measured volume of 5.4 mm^3^ (**B2**).

Mastoid pneumatization was classified as pneumatized, diploic, or sclerotic based on HRCT findings [12,13] (Figure 3).

### 2.3. Statistical Analysis

All statistical analyses were performed using SPSS software (version 22.0; IBM Corp., Armonk, NY, USA). Normality of the data was assessed using the Shapiro–Wilk test. Continuous variables were reported as mean ± standard deviation (SD) or median with interquartile range (IQR), depending on distribution, while categorical variables were expressed as counts and percentages. Comparisons between two groups were performed using two-tailed independent-samples *t*-tests, Mann–Whitney U tests, or chi-square (χ^2^) tests, as appropriate. For three-group comparisons, one-way ANOVA or Kruskal–Wallis tests were applied, followed by Bonferroni or Tukey’s post hoc tests. Multivariable linear regression was used to identify independent predictors of STR volume, adjusting for age, sex, disease type, and mastoid pneumatization. For the regression model, disease type was categorized into CSOM and CNSOM, with the control group serving as the reference category. Similarly, mastoid pneumatization was categorized into diploic and sclerotic types, using the pneumatized mastoid as the reference category. Interobserver and intraobserver reliability were assessed using intraclass correlation coefficients (ICCs; two-way, absolute agreement, single measures) for continuous variables and Cohen’s kappa coefficients for categorical variables. In addition, Bland–Altman analyses were performed to visualize agreement between repeated measurements. A *p*-value < 0.05 was considered statistically significant.

## 3. Results

The study cohort comprised 187 patients (222 ears) divided into the CSOM group (*n* = 67), the CNSOM group (*n* = 85), and the control group (*n* = 35, all bilateral). The demographic details are shown in Table 1, with no significant differences in age (ANOVA, *p* = 0.42) or sex distribution (chi-square, *p* = 0.28) among the groups. The median duration of illness was 5 [3–8] years in the CSOM group and 5 [3–7] years in the CNSOM group, with no statistically significant difference (*p* = 0.617).

Mastoid pneumatization differed significantly among the three groups (χ^2^ = 96.46, df = 2, *p* < 0.001), with both CSOM and CNSOM patients showing markedly reduced pneumatization compared to controls. In the pairwise post hoc analyses, no statistically significant differences were observed between CSOM and CNSOM (χ^2^ = 5.13, df = 2, *p* = 0.077) (Table 2). In the control group, analysis of 70 ears from 35 patients revealed no significant difference between right and left mastoid pneumatization (Bowker symmetry test, *p* = 0.18).

**Figure 4:**
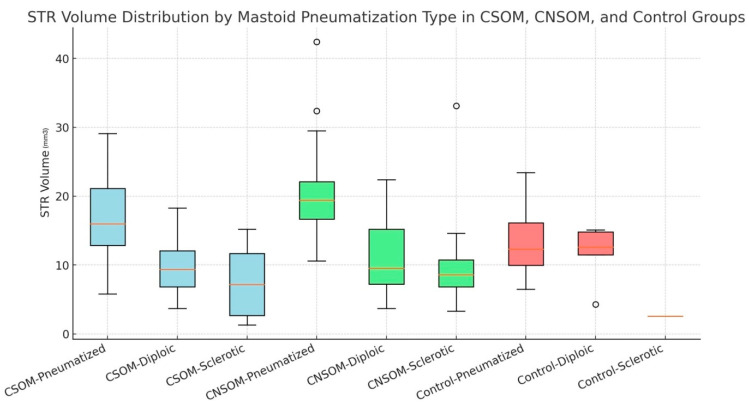
STR volume distribution (mm^3^) by mastoid pneumatization type in CSOM (Blue), CNSOM (Green), and control groups (Light red). White circles represent statistical outliers. STR: supratubal recess, CSOM: chronic suppurative otitis media, CNSOM: chronic non-suppurative otitis media.

STR volume showed no significant difference among the CSOM, CNSOM, and control groups (*p* = 0.751). In contrast, a significant difference was observed according to the mastoid pneumatization pattern (*p* < 0.001). Pairwise comparisons with Bonferroni correction demonstrated significantly higher STR volumes in pneumatized ears compared to both diploic and sclerotic ears, whereas no statistically significant difference was observed between the diploic and sclerotic groups after adjustment (Table 3, Figure 4). Beyond volume, STR length, width, and height were also significantly greater in pneumatized mastoids compared to diploic and sclerotic types (*p* < 0.001) (Table 3). No significant difference in STR volume was observed among age groups (18–30, 31–50, 51–65) (*p* > 0.05 for all comparisons) (Table 3). Although univariate analysis revealed no significant differences in STR volumes among the CSOM, CNSOM, and control groups, this unadjusted comparison was heavily masked by baseline differences in mastoid pneumatization (Table 2). Because pneumatization is the primary determinant of STR volume, disease type was a priori included in the multivariable regression model to properly adjust for this confounding effect alongside age and sex.

**Figure 5:**
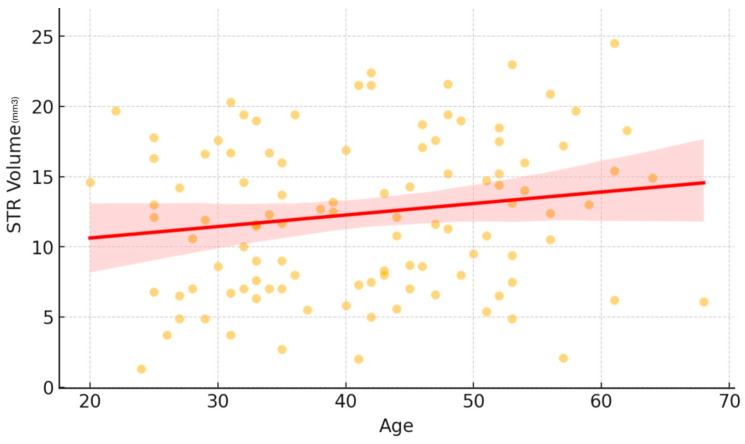
Relationship between age and STR volume (mm^3^). STR: supratubal recess. The yellow dots represent individual data points, the solid red line indicates the linear regression line, and the shaded light red area represents the 95% confidence interval.

The multivariable linear regression model incorporating mastoid pneumatization type, disease group, sex, and age was statistically significant (*F*(6, 215) = 18.078, *p* < 0.001) and explained 33.5% of the variance in STR volume (R^2^ = 0.335, adjusted R^2^ = 0.317). In multivariable regression analysis, we found that mastoid pneumatization type and disease group were independently associated with STR volume, while age and sex did not reach statistical significance (Table 4, Figure 5).

In the complementary regression model focusing on STR dimensions, STR height emerged as the strongest predictor of STR volume (B = 2.784, β = 0.430, *p* < 0.001), followed by STR length (B = 1.950, β = 0.298, *p* < 0.001) and STR width (B = 2.612, β = 0.251, *p* < 0.001). This model explained 59.2% of the variance in STR volume (R^2^ = 0.592), with no evidence of multicollinearity (VIF < 2).

Intraobserver reliability was high. The ICC for continuous data was 0.88 (95% CI: 0.82–0.94), indicating good agreement, while Cohen’s kappa coefficient for categorical data showed similarly strong consistency (κ = 0.84; 95% CI: 0.73–0.92). Interobserver reliability ranged from moderate to good. Cohen’s kappa again indicated strong consistency for categorical data (κ = 0.79; 95% CI: 0.66–0.88), whereas ICC values for STR length, width, height, and volume were 0.64 (95% CI: 0.51–0.80), 0.72 (95% CI: 0.54–0.84), 0.78 (95% CI: 0.64–0.91), and 0.82 (95% CI: 0.74–0.92), respectively.

## 4. Discussion

This study investigated the relationship between mastoid pneumatization and age-related changes in STR volume among patients with COM using 3D HRCT. Our results indicate that STR volume is strongly influenced by mastoid pneumatization. Furthermore, in the multivariable analysis, both CSOM and CNSOM were independently associated with a larger STR volume. Although age did not exert a statistically significant effect, a borderline trend toward increased STR volume in adulthood was observed. Importantly, 3D HRCT volumetry provided a refined anatomical characterization of the supratubal recess, and reliability analyses confirmed good intraobserver agreement and moderate-to-good interobserver consistency.

Given the STR’s close anatomical relationship with critical structures, comprehensive knowledge of its morphological variations is essential for safe otologic surgery [1,2,14]. The STR’s deep and narrow configuration creates significant surgical challenges, as restricted access and poor visualization may lead to incomplete treatment and higher complication risks [7,8,9]. For optimal preoperative planning, HRCT of the temporal bone is recommended to evaluate STR pneumatization and anatomy.

Tono et al. reported that during the late fetal stage, the bony Eustachian tube (ET) expands upward toward the geniculate fossa, forming the dome-shaped supratubal recess (STR). STR pneumatization is thought to occur primarily via the ET and continues to enlarge throughout childhood [5]. Whereas Hong et al. focused on ET morphology, Palva and Inanlı investigated the role of the tensor fold in STR development [3,4,7]. Hong et al. further demonstrated that impaired ET-to-STR ventilation may contribute to reduced STR volume and epitympanic mucosal pathology [4]. Alternatively, Inanlı et al. proposed that STR aeration is maintained via the tympanic isthmus and may be compromised in the presence of an intact tensor fold [7]. Although we did not directly evaluate the ET or tensor fold, the strong correlation between mastoid pneumatization and STR volume observed in our study underscores the importance of posterior aeration pathways in adults. Moreover, across all three groups—controls, CSOM, and CNSOM—STR volume was consistently highest in pneumatized mastoids, independent of disease type. In multivariable analyses, mastoid pneumatization emerged as the strongest independent correlate of STR volume. However, our regression model explained 33.5% of the variance in STR volume, indicating moderate explanatory power and suggesting stronger hidden determinants. Anatomical variables (mastoid air cell system volume, ET angle/patency), clinical factors (disease duration, recurrent otitis, childhood infection history), and weaker demographic influences (smoking, BMI) may all contribute. Larger adult cohorts are needed to validate these findings and clarify the interplay between STR, ET, tensor fold, and mastoid pneumatization.

The relationship between pneumatization patterns and surgical anatomy remains clinically significant. Previous studies have correlated extensive pneumatization with larger sinus tympani and STR volumes [14,15]. Our findings also demonstrate that mastoid pneumatization is strongly associated with STR volume, with the largest volumes observed in well-pneumatized temporal bones. A critical surgical consideration is that a deep STR may require more extensive bone removal and greater reduction in the facial ridge, and in well-pneumatized mastoids the facial nerve is at a heightened risk of iatrogenic injury [14,16]. Inadequate exploration or blind dissection of this region has been linked to an increased likelihood of residual or recurrent disease because of hidden recesses and carries a substantial risk of complications, especially in advanced COM patients [7,9,17]. In such cases, being prepared to perform an extended atticotomy or to employ endoscope-assisted approaches may help optimize surgical exposure. These collective findings highlight the importance of meticulous preoperative evaluation of the STR, particularly in well-pneumatized temporal bones. Incorporating detailed STR assessment into radiological practice may provide additional anatomic information that facilitates more accurate preoperative planning and potentially improves surgical outcomes.

According to the literature, mastoid pneumatization plays a crucial role in middle ear ventilation and disease pathogenesis. The environmental theory suggests that chronic otitis media (e.g., serous, purulent) may inhibit mastoid pneumatization, whereas the genetic theory proposes that inherently poor mastoid aeration contributes to middle ear pathologies [18,19,20]. Consistent with previous reports, our findings demonstrated significantly better mastoid pneumatization in controls (88.6% pneumatized mastoids) compared to disease groups, with no significant right–left asymmetry in controls. To investigate inflammatory influences, we compared three cohorts: CSOM, CNSOM, and controls. No significant differences in mastoid pneumatization or STR volume were observed between CSOM and CNSOM in the univariable analysis. However, in the multivariable regression model, both CSOM and CNSOM emerged as independently associated with a larger STR volume. The clinical meaning of this result should be interpreted with caution. Earlier radiographic studies reported no differences in epitympanic recess volumes between unilateral or bilateral COM and healthy controls [21]. Therefore, more comprehensive, large-scale studies are warranted to further elucidate these anatomical relationships and validate their clinical implications.

Although the development of the STR in childhood has been well documented, its age-related morphological changes in adulthood remain poorly understood. Hong et al. examined the relationship between age and STR morphology, reporting that STR-W gradually decreases with age, STR-L increases until age 18 and then stabilizes, while STR-H remains unchanged. They also proposed that STR aeration is closely related to tympanic orifice caliber and the functional status of the ET and middle ear [4]. Their results, however, were derived from a limited number of adult specimens (12 temporal bones from 8 adults), which may restrict generalizability. Our comprehensive analysis of 222 temporal bones from 187 adults demonstrated stable STR-L and STR-W, with a nonsignificant trend toward increasing STR-H and STR volume with advancing age. Nonetheless, as our results are derived from cross-sectional associations, longitudinal studies are needed to confirm potential post-maturational changes and their clinical relevance, and to further elucidate alterations in epitympanic ventilation pathways. Additionally, incorporating pediatric cohorts across different age ranges (e.g., 0–3 years, 4–8 years, 9–18 years) and comparing them with adult data would allow for a more comprehensive understanding of STR development throughout life.

Our findings suggest that HRCT-based volumetric analysis is a reliable method for assessing STR dimensions. While previous studies have explored the relationship between mastoid pneumatization and middle ear structures, none have specifically examined its impact on STR volume using high-resolution three-dimensional volumetric analysis [2,4,9,12,15]. Traditional two-dimensional CT measurements are common but prone to inaccuracies due to head positioning. In contrast, 3D volumetric assessments offer a more precise and comprehensive evaluation. Accurate STR volume measurement could provide valuable insights into middle ear pathophysiology and may also enhance surgical planning, particularly for endoscopic ear procedures. Given the importance of visualizing deep recesses such as the STR, volumetric analysis can aid in surgical navigation. However, manual segmentation, despite demonstrating high interobserver and intraobserver reliability in the present study, remains time-consuming and potentially prone to operator-dependent variability. By comparison, semi-automatic and automatic approaches may yield reproducible volumetric measurements with minimal user intervention, thereby offering substantially shorter processing times and reduced operator dependency [22]. Future studies incorporating semi-automated or AI-assisted volumetric methods may further validate and expand the clinical applicability of STR volumetric analysis.

This study has several limitations that should be considered. First, the retrospective design may introduce selection bias, despite our rigorous inclusion criteria, particularly since the control group was composed of cochlear implant candidates, who may not fully represent a normal otologic population. Second, while 3D HRCT provides excellent anatomical detail of STR morphology, it cannot assess functional aspects of middle ear ventilation that may influence pneumatization patterns. Additionally, although intra- and interobserver variability metrics were calculated and demonstrated acceptable reliability, the manual nature of volume segmentation still represents a potential methodological limitation. Future studies could benefit from automated or semi-automated segmentation techniques to further reduce observer-dependent variability. Third, as a cross-sectional study, our design does not allow for the assessment of temporal changes within the same individuals; however, no statistically significant age-related differences were identified in our cohort. Our findings specifically address non-cholesteatomatous COM, as we excluded cholesteatoma cases to avoid confounding by osseous erosion. While this exclusion strengthens the internal validity of our pneumatization measurements, it means our results cannot be directly extrapolated to cholesteatomatous COM evaluation. Future investigations should examine surgical outcomes relative to STR pneumatization patterns and conduct prospective studies of cholesteatoma cases with STR involvement. Despite these limitations, our study provides crucial data on STR anatomy in COM and its age-related changes in adulthood, establishing a foundation for future research on pneumatization patterns.

## 5. Conclusions

STR volume is strongly influenced by mastoid pneumatization and independently associated with the presence and type of chronic otitis media, whereas age-related changes in adulthood and sex are negligible. Three-dimensional HRCT-based volumetric assessment appears to be a valuable tool for evaluating STR morphology, and may facilitate more comprehensive anatomical and radiological evaluations. Further studies integrating functional data and longitudinal designs are warranted to validate and expand upon these findings.

## Figures and Tables

**Table 1:** Patient demographics and clinical data.

	Patients, n	Mean Age (±SD)	Female, n (%)	Male, n (%)	Left Ear, n	Right Ear, n
CSOM group	67	41.4 ± 11.02	45 (67.2%)	22 (32.8%)	40	27
CNSOM group	85	39.44 ± 12.18	50 (58.8%)	35 (41.2%)	39	46
Control group	35	43.51 ± 10.98	18 (51.4%)	17 (48.6%)	35	35
		*p* = 0.42 *	*p* = 0.28 **			

CSOM: chronic suppurative otitis media, CNSOM: chronic non-suppurative otitis media, n: number, *: one-way ANOVA, **: chi-square test.

**Table 2:** Mastoid pneumatization patterns in CSOM, CNSOM, and Controls.

Mastoid Pneumatization Type	CSOM Group ^a^ n (%)	CNSOM Group ^b^ n (%)	Control Group ^c^ n (%)	*p* ^a vs. b,^*	*p* ^a vs. c,^*	*p* ^b vs. c,^*	*p* ^all groups,^*
Pneumatized	31 (46.3%)	19 (22.4%)	62 (88.6%)	0.077	<0.001	<0.001	<0.001
Diploic	19 (28.4%)	32 (37.6%)	6 (8.6%)				
Sclerotic	17 (25.3%)	34 (40.0%)	2 (2.9%)				

CSOM: chronic suppurative otitis media, CNSOM: chronic non-suppurative otitis media, n: number, *: chi-square test, ^a^: CSOM Group, ^b^: CNSOM Group, ^c^: Control Group.

**Table 3:** Comparison of STR volume and dimensions according to disease subgroups, mastoid pneumatization patterns, and age groups.

Disease Type	CSOM Group ^a^ (n = 67)	CNSOM Group ^b^ (n = 85)	Control Group ^c^ (n = 70)	*p* ^a vs. b,^*	*p* ^a vs. c,^*	*p* ^b vs. c,^*	*p* ^all groups,^**
STR Volume (mm^3^) (mean ± SD)	12.46 ± 6.39	12.51 ± 7.19	13.18 ± 4.90	1.000	1.000	1.000	0.751
**Mastoid pneumatization type**	**Pneumatized ^d^ (n = 112)**	**Diploic ^e^** **(n = 57)**	**Sclerotic ^f^** **(n = 53)**	***p*** ^d vs. e,^*	***p*** ^d vs. f,^*	***p*** ^e vs. f,^*	***p*** ^all groups,^**
STR Volume (mm^3^) (median [IQR])	15.05 [11.43–19.35]	9.5 [6.85–14.55]	8.0 [6.0–10.8]	<0.001	<0.001	0.019	<0.001
STR Length	3.91 ± 0.92	3.27 ± 0.81	2.78 ± 0.67	<0.001	<0.001	0.007	<0.001
STR Width	2.67 ± 0.53	2.36 ± 0.61	2.09 ± 0.54	0.002	<0.001	0.032	<0.001
STR Height	3.75 ± 0.95	3.12 ± 0.89	3.00 ± 0.85	<0.001	<0.001	0.747	<0.001
**Age groups**	**18–30 ^g^**	**31–50 ^h^**	**51–65 ^i^**	***p*** ^g vs. h,^*	***p*** ^g vs. i,^*	***p*** ^h vs. i,^*	***p*** ^all groups,^**
STR Volume (mm^3^) (median [IQR])	11.3 [7.18–16.55]	11.6 [8.0–16.7]	12.2 [7.9–16.05]	0.585	0.659	0.942	0.855

CSOM: chronic suppurative otitis media, CNSOM: chronic non-suppurative otitis media, n: number, STR: Supratubal recess, SD: Standard deviation, IQR: Interquartile range, ^a^: CSOM Group, ^b^: CNSOM Group, ^c^: Control Group, ^d^: pneumatized, ^e^: diploic. ^f^: sclerotic, ^g^: 18-30 years, ^h^: 31-50 years, ^i^: 51-65 years, *: independent sample *t*-test, **: one-way ANOVA, *: Mann–Whitney U test, **: Kruskal–Wallis test, *: Mann–Whitney U test, **: Kruskal–Wallis test.

**Table 4:** Multivariable linear regression analysis of factors associated with STR volume (mm^3^).

Variable	B (Unstandardized)	Std. Error	Β (Standardized)	*t*	*p*-Value	95% CI
Age	0.069	0.031	0.126	2.117	0.053	−0.001 to 0.130
Sex	0.627	0.720	0.049	0.870	0.385	−0.793 to 2.046
Pneumatization type (Diploic) *	−7.448	0.959	−0.519	−7.770	<0.001	−9.337 to −5.558
Pneumatization type (Sclerotic) *	−9.778	1.010	−0.665	−9.676	<0.001	−11.769 to −7.786
Disease type (CSOM) **	3.006	0.970	0.220	3.099	0.002	1.094 to 4.918
Disease type (CNSOM) **	5.367	1.031	0.416	5.207	<0.001	3.335 to 7.399

* Reference category: Pneumatized mastoid. ** Reference category: Control group. CI: Confidence interval.

## Data Availability

The data that support the findings of this study are available on request from the corresponding author.

## Abbreviations

STR	Supratubal recess
HRCT	High-resolution computed tomography
CSOM	Chronic suppurative otitis media
CNSOM	Chronic non-suppurative otitis media
3D	Three-dimensional
COM	Chronic otitis media
ICCs	Intraclass correlation coefficients
STR-W	STR Width
STR-L	STR Length
STR-H	STR Height
SD	standard deviation
IQR	Interquartile range
ET	Eustachian tube
